# The impact of premorbid and current intellect in schizophrenia: cognitive, symptom, and functional outcomes

**DOI:** 10.1038/npjschz.2015.43

**Published:** 2015-11-04

**Authors:** Ruth Wells, Vaidy Swaminathan, Suresh Sundram, Danielle Weinberg, Jason Bruggemann, Isabella Jacomb, Vanessa Cropley, Rhoshel Lenroot, Avril M Pereira, Andrew Zalesky, Chad Bousman, Christos Pantelis, Cynthia Shannon Weickert, Thomas W Weickert

**Affiliations:** 1 School of Psychiatry, University of New South Wales, Sydney, NSW, Australia; 2 Neuroscience Research Australia, Randwick, Sydney, NSW, Australia; 3 Department of Psychiatry, University of Melbourne, Parkville, VIC, Australia; 4 Northern Psychiatry Research Centre, North Western Mental Health, Melbourne Health, Victoria, Australia; 5 Schizophrenia Research Institute, Sydney, NSW, Australia; 6 Molecular Psychopharmacology Laboratory, The Florey Institute of Neuroscience and Mental Health, Parkville, VIC, Australia

## Abstract

**Background:**

Cognitive heterogeneity among people with schizophrenia has been defined on the basis of premorbid and current intelligence quotient (IQ) estimates. In a relatively large, community cohort, we aimed to independently replicate and extend cognitive subtyping work by determining the extent of symptom severity and functional deficits in each group.

**Methods:**

A total of 635 healthy controls and 534 patients with a diagnosis of schizophrenia or schizoaffective disorder were recruited through the Australian Schizophrenia Research Bank. Patients were classified into cognitive subgroups on the basis of the Wechsler Test of Adult Reading (a premorbid IQ estimate) and current overall cognitive abilities into preserved, deteriorated, and compromised groups using both clinical and empirical (*k*-means clustering) methods. Additional cognitive, functional, and symptom outcomes were compared among the resulting groups.

**Results:**

A total of 157 patients (29%) classified as ‘preserved’ performed within one s.d. of control means in all cognitive domains. Patients classified as ‘deteriorated’ (*n*=239, 44%) performed more than one s.d. below control means in all cognitive domains except estimated premorbid IQ and current visuospatial abilities. A separate 138 patients (26%), classified as ‘compromised,’ performed more than one s.d. below control means in all cognitive domains and displayed greater impairment than other groups on symptom and functional measures.

**Conclusions:**

In the present study, we independently replicated our previous cognitive classifications of people with schizophrenia. In addition, we extended previous work by demonstrating worse functional outcomes and symptom severity in the compromised group.

## Introduction

Cognitive impairment is a characteristic of schizophrenia^1^ associated with negative symptom severity,^2,3^ and functional impairment.^4,5^ Evidence suggests distinct neuropsychological profiles of cognitive deficits in schizophrenia,^2,6–8^ that may reflect differing courses of abnormal neurodevelopment.^9^ Longitudinal studies have revealed a link between lower childhood IQ and schizophrenia.^10^ However, premorbid intellectual impairment is associated with many psychiatric diseases^11^ and not all people with schizophrenia display intellectual impairment.^6,12^

Many patients with schizophrenia display intact levels of crystallized intelligence on Wechsler Adult Intelligence Scale (WAIS) tests^13^ such as in general knowledge (e.g., Vocabulary), and display impairment on tests of more fluid intelligence, such as verbal abstract concepts (e.g., Similarities) and working memory (e.g., Arithmetic).^14^ Tests of crystallized verbal intelligence^15^ can act as ‘hold tests’ that assess premorbid IQ.^16,17^ Weickert *et al*.^6^ classified patients with schizophrenia into three cognitive subgroups on the basis of estimated premorbid and current IQ using both empirical (*k*-means clustering) and clinical (falling within specific ranges) grouping methods. They found a deteriorated group (50%), exhibiting decline (⩾10 points) from an estimate of premorbid IQ (Wide Range Achievement Test^18^) to current IQ; a compromised group (25%), with low premorbid and low current IQ estimates; and a preserved group (25%), with near to above average premorbid and current IQ estimates. Those patients in the compromised group showed widespread cognitive deficits, whereas patients in the preserved group had deficits restricted to attention and executive function and the deteriorated group displayed memory deficits in addition to those deficits shown in the preserved group. The authors^6^ suggested that these results may reflect distinct subtypes of disease progression, with the compromised group showing early global deficits, the deteriorated group showing deficits possibly associated with pathology at the time of illness onset, and the preserved group, which appears to remain relatively cognitively intact.

Much subsequent work has supported cognitive subtype classifications in schizophrenia.^2,7,8,19,20^ Among first episode patients, those with preserved intellect differed from other patients on spatial working memory, verbal memory, and executive function tests.^8,20^ Patients in the preserved group displayed significantly better performance on tests of executive function (i.e., verbal fluency, spatial working memory) and attention (i.e., continuous performance test), but show similar cognitive impairments relative to other patients on a test of processing speed or verbal working memory.^7,19^ Although there is no consensus regarding which cognitive tests delineate these subgroups; overall, these findings provide further support for the existence of distinct neuropsychological subgroups in schizophrenia. Research thus far has been limited by relatively small samples sizes (<130 people with schizophrenia) and has usually used clinical cutoffs to create groups, rather than data-driven procedures that may be more sensitive to variations in cognitive performance. There has also been limited examination of cognitive subgroup symptom and functional profiles, which is of relevance given their documented relationship to cognition in schizophrenia.^3,5^

The aims of the present study were to provide independent replication of these IQ subgroups^6^ using both clinical and empirical clustering in a relatively large community-based cohort and to assess the extent to which the different cognitive subgroups display negative symptoms and functional deficits. We hypothesized that: (1) relative to healthy controls, ~25% of patients with schizophrenia would display compromised premorbid and current cognitive function and global deficits in neuropsychological function; (2) approximately 50% of patients with schizophrenia would display deterioration from premorbid to current cognitive function and deficits in executive function, memory, and attention; (3) approximately 25% of patients would display preserved premorbid and current cognitive function and would have cognitive deficits limited to measures of executive function and attention; and (4) patients in the compromised group would display the most severe deficits on both premorbid and current function as well as greater severity of negative symptoms.

## materials and Methods

### Participants

Clinical and cognitive data for 534 patients with schizophrenia and 635 healthy controls were obtained from the Australian Schizophrenia Research Bank (ASRB), a resource of research data collected across five Australian states and territories.^21^ Patients had a confirmed diagnosis of schizophrenia (*n*=448) or schizoaffective disorder (*n*=86) using DSM-IV criteria from the Diagnostic Interview for Psychosis (DIP).^22^ All participants (ages 18–65 years) were fluent in English. Childhood onset was not an exclusion factor; however, only adults were recruited and only 0.7% of the total patient sample met criteria for childhood onset, i.e., having illness onset between 11 and 12 years of age in our sample. Participants with a history of: organic brain disorder; serious brain injury; an IQ<70; or electroconvulsive therapy or current substance dependence were excluded. Healthy controls with a personal or family history of psychosis or bipolar I disorder were also excluded. See Table 1 for demographic characteristics of the participants. Ethics approval for data collection (HNE HREC 08/12/17/5.20, HREC/08/HNE/438, SSA/09/HNE/23) and analysis (Human Research Ethics Advisory Panel of the University of New South Wales, HREA UNSW 2014-7-53) was provided and all participants gave informed consent.

### Premorbid IQ

Premorbid IQ was assessed using the Wechsler Test of Adult Reading (WTAR),^15^ a marker of premorbid intellectual ability,^13,16,17^ which assesses correct pronunciation of English words (mean=100, s.d.=10). WTAR scores have been shown to remain stable across repeated testing, compared with variation in tests of current ability.^16^

### Current cognitive function

The Repeatable Battery for the Assessment of Neuropsychological Status (RBANS),^23^ with demonstrated validity in schizophrenia^24^ and age-adjusted Index scores, was used to assess performance in the cognitive domains of immediate and delayed verbal memory, attention, language, and visual–spatial perception. Tests of working memory (i.e., WAIS-III Letter Number Sequencing, age-adjusted scaled scores)^13^ and executive function/language (i.e., Controlled Oral Word Association Test (COWAT), age- and gender-adjusted scores^25^) were also administered. Index scores were converted to *z*-scores relative to the healthy control means and s.d.

### Current global cognitive ability estimate

The Vocabulary—Matrix Reasoning dyad of the Wechsler Abbreviated Scale of Intelligence^26^ was administered in the ASRB as a measure of current IQ but was not included in this analysis as it has been shown to inaccurately inflate IQ scores^27^ and to be insensitive to cognitive decline.^28^ A measure of crystallized intelligence (e.g., Vocabulary^16^) is not appropriate in a group in which intellectual performance declines over time. Thus, neuropsychological tests highly correlated with current IQ, RBANS Immediate Memory and Attention and WAIS-III Letter Number Sequencing (LNS), were converted to *z*-scores and the *z*-scores were averaged for each participant to obtain an estimate of current global cognitive function. Among patients with schizophrenia, RBANS Immediate Memory and Attention are more strongly correlated with IQ (*r=*0.60 and *r=*0.73, respectively),^29^ and are impaired relative to language and visuospatial tests.^24^ In general, WAIS-III immediate memory is also more strongly associated with IQ than delayed memory.^30^ LNS is strongly correlated with IQ^13^ and it has differentiated between patients with schizophrenia displaying preserved and deteriorated cognitive function.^7,20^

### Classification of groups

Patients were empirically clustered into groups as per our original report^6^ and these groups were used in further analyses to characterize the subgroups. Empirical clustering methods reduce subjectivity and are more reliable than clinical groupings at ensuring maximum proximity to cluster centers and distance from other clusters for each measurement.^31^ *Z*-scores (based on the healthy control means and s.d.) for the WTAR, WAIS-III LNS, and RBANS Attention and Immediate memory Index scores were entered into a tree clustering hierarchical cluster analysis using squared Euclidean distances (progressively placing greater weight in objects that are further apart) to initially define the distance between items and form the clusters along with complete linkage (using the furthest neighbor rule to determine when two clusters are similar enough to be linked) to determine the distances between clusters. The resulting dendrogram (a tree diagram) was inspected by three raters (RW, DW, TWW) who reached consensus on a three group solution, which was also consistent with our previous work.^6^ We next entered these data into a *k*-means clustering analysis with the number of clusters equal to three to identify three clusters with the greatest possible distinction. Patients were also separately categorized clinically into three groups based on decline from estimated premorbid IQ to determine the extent to which we could replicate our previous clinical categorization strategy in a larger independent sample (see Supplementary Methods for more details).

### Symptom severity and functional outcome assessment

Negative symptoms were measured using the Scale for Assessment of Negative Symptoms (SANS).^32^ Positive symptom severity estimates were calculated from the total score of lifetime or present hallucination and delusion ratings from the DIP^22^ (items 49–53 and 58–64, respectively^33^). Additional demographic factors and items from the DIP (items 9, 13–15, and 17)^22^ and the Global Assessment of Functioning (GAF) scores^34^ were used as measures of premorbid and current functioning.

### Statistical analyses

One-way analysis of variances (ANOVAs) and *χ*^2^-analyses were performed on demographic and clinical variables. A series of four one-way ANOVAs were performed, using the three empirically derived patient cognitive groups along with healthy controls as a grouping factor, and gender as a separate grouping factor on each cognitive variable (significance level *P*=0.05, Bonferroni adjustment: main effects *P*=0.0125; planned *post hoc* comparisons *P*=0.002). For non-normally distributed variables (RBANS language and delayed memory) nonparametric Kruskal–Wallis tests were used to compare mean ranks among the groups. A MANOVA was performed to test for group differences on the SANS (significance level *P*=0.05, univariate Bonferroni adjustment *P*=0.01). Planned *post hoc* tests were performed on significant results to test for differences among groups (Bonferroni adjusted, *P*=0.005). Non-parametric Kruskal–Wallis tests were used to test for differences among groups in positive symptoms with planned *post hoc* comparisons on significant results (Bonferroni adjusted, *P*=0.016). Cohen’s *d* effect sizes were calculated.^35^ A series of *χ*^2^-tests for general functioning and adjustment items from the DIP and a one-way ANOVA for the GAF assessed differences in functioning among groups (Bonferroni adjustment *P*=0.004 for univariate main effects and *P*=0.003 for planned *post hoc* comparisons).

## Results

### Cluster analyses

**Figure 1:**
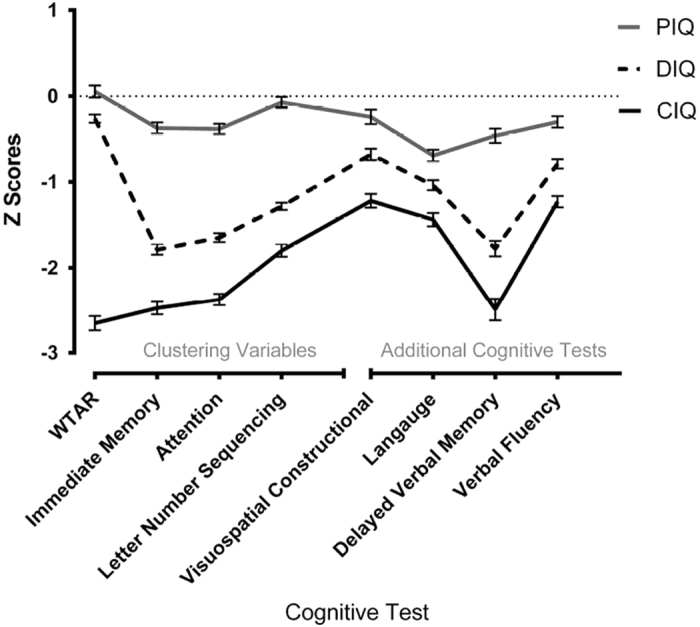
Cognitive domain profiles of the preserved, deteriorated, and compromised patient groups. Error bars indicate s.e. Attention, attention Index of RBANS; CIQ, compromised intellectual function group; DIQ, deteriorated intellectual function group; IM, immediate memory Index of Repeatable Battery for the Assessment of Neurospychological Status (RBANS); LNS, letter number sequencing subtest (WAIS-III); PIQ, preserved intellectual function group; WTAR, Wechsler test of adult reading.

The empirical clustering method produced three groups of patients displaying: (1) a decline from premorbid cognitive functioning (‘deteriorated’ group, 44%); (2) compromised intellectual functioning (‘compromised’ group, 26%); and (3) preserved intellectual functioning (‘preserved’ group, 29%; Figure 1). Supplementary Figure S1 shows the relationships between WTAR scores and current global cognitive ability measures for each individual in each group. Patients in the deteriorated group displayed average to high premorbid (WTAR) and low current global cognitive ability estimates, whereas those in the compromised group displayed low scores on all measures and those in the preserved group displayed a pattern most similar to controls. Cross tabulation between clinical and empirical grouping methods indicated 89–91% agreement, demonstrating that the empirical clusters were clinically meaningful and corresponded to patients being either in or out of the normal range (> or <1 s.d. from control means).

### Demographic results

Table 2 lists demographic characteristics of patients in the empirically derived groups and healthy controls. There were no significant differences among groups in illness duration. There was a significant difference among patient groups in age of onset. The preserved group had a significantly older age of onset than the deteriorated group by 1.7 years; however, this approximately 7% difference had a small effect size (*d*=0.28). There were significant differences among groups in age, with the preserved the oldest and deteriorated the youngest patients, and healthy controls significantly older than all but the preserved group. There were significant differences among all the groups in years of education, which was significantly correlated with WTAR scores (*r*=0.46, *P*<0.001). There were significant differences among patient groups and controls in gender ratios, with more male than female patients and more female than male controls and significantly more male than female patients in compromised relative to preserved groups. The majority of patients (88%) were currently receiving antipsychotic medication, with most receiving second-generation antipsychotics. See Supplementary Table S1 for frequencies of antipsychotic combinations amongst patients in the cognitive groups.

For comparison between grouping procedures, Supplementary Table S2 lists demographic characteristics of patients in the clinically derived groups. Significant differences among the groups were similar to the empirically derived groups, except that there was no significant difference between compromised and preserved groups in age; a significant difference between compromised and deteriorated groups in age of onset (deteriorated group with a younger onset age); and no significant difference between compromised and preserved groups in gender ratios.

### Additional neuropsychological measures

There were significant differences among groups in all additional cognitive domains and significant differences between all pairwise comparisons from *post hoc* LSD tests after Bonferroni correction (*P*<0.002) except for pairwise comparisons between the preserved group and controls in visuospatial performance. See Table 3 for group means and s.d. As the nonparametric results were similar to those produced with parametric tests, the ANOVA results are displayed in Table 3 along with interactions between the groups. There were significant main effects of cognitive group and gender (with females performing better than males) on language and visuospatial construction. For delayed memory, there were significant main effects of cognitive group (*χ*^2^=475.50, *P*<0.001) and gender (*χ*^2^=27.58, *P*<0.001), with females performing better than males. There was a significant interaction between group and gender in delayed verbal memory. *Post hoc* tests indicated that among patients there was no significant interaction between cognitive groups and gender, F(2,528)=0.256, *P*=0.775; however, there was a significant interaction between gender and diagnosis, F(1,1157)=16.82, *P*<0.001, with male patients performing significantly worse than female patients, *t*(533)=7.92, *P*<0.001, but no significant difference between healthy men and women, *t*(626)=0.85, *P*=0.329. There were no interactions between group and gender in any other cognitive variable. Cohen’s *d* effect sizes are shown for pairwise comparisons to facilitate interpretation. The compromised group performed markedly worse than controls in all domains, indicated by large effect sizes (all *d*>0.8). The deteriorated group displayed large effect sizes compared to controls in all domains except visuospatial ability. The preserved group did not display any large effect sizes compared with controls in any domain. See Supplementary Table S3 for group sizes, means, and medians on additional neuropsychological measures based on the clinical clustering strategy.

### Symptom severity

There were significant differences among the groups in relation to negative symptom severity in the domains of avolition–apathy, affective flattening and anhedonia–asociality, see Table 4. Patients in the compromised group displayed significantly more negative symptoms than those in the preserved group for ten items with medium effect sizes (*d*=0.3–0.5). The compromised group did not display significantly different symptom severity relative to the deteriorated group. There was a significant difference between the preserved and other groups in physical anergia with compromised and deteriorated groups displaying more symptoms than the preserved group. Patients in the compromised group reported significantly more hallucinations than patients in the preserved group (*d*=0.47; see Table 4). There were no differences in lifetime reported hallucinations among groups. Patients in the preserved group reported significantly more lifetime delusions than patients in the compromised group (*d*=0.35). There were no other significant differences among groups in reference to delusions. These group differences in negative symptoms, hallucinations, and delusions generally represent small to medium effect sizes and may not be clinically relevant.

### Functional outcome

Chi-square analyses of levels of functioning revealed significant differences among groups (Table 4). When compared to either the deteriorated or preserved groups, the compromised group were more likely to be: (1) unemployed prior to onset; (2) to currently have fewer friends and be recently unemployed; and (3) were rated as having lower global functioning on the GAF. Relative to the preserved group only the compromised group reported a chronic course without periods of recovery between episodes. The deteriorated and preserved groups were not significantly different on any functional variables. There were no significant differences between any groups in relation to other functional measures.

## Discussion

Our analysis of a relatively large community cohort using both clinical and empirical methods, provides independent replication of previous research showing three distinct cognitive phenotypes in schizophrenia.^6^ Forty-four percent of the patients displayed deterioration from estimated premorbid IQ, with putatively intact cognitive function during periods of development crucial for the formation of crystallized intelligence, followed by a decline in mostly fluid intelligence, to well below-normal levels. The remainder of the patients did not show clear decline and were divided into the compromised group, with low premorbid and current intellectual function, and the preserved group, with average or above premorbid and current global cognitive ability. All subgroups displayed significant differences in almost all additional cognitive domains. These groupings are consistent with previous research in diverse samples,^2,6–8,19,20^ including imaging and neuropsychological results in early psychosis,^36^ indicating that these subtypes may represent different cognitive trajectories underlying manifestation of the illness, as opposed to effects of antipsychotics and/or chronic illness and hospitalization.

Consistent with previous studies, patients in the compromised group performed below the normal range (greater than one s.d. below control means), with effect sizes greater than one, compared with healthy controls, in all domains.^6,7^ This included impaired performance on visual processing tests, possibly indicating more widespread impairment. Impaired visuospatial tests included line orientation, which is associated with occipital and parietal abnormalities^37^ and does not typically differentiate patients with schizophrenia from healthy controls;^38^ and figure copy and recall, which has been associated with more widespread cortical atrophy in more advanced Alzheimer’s disease.^39^ Given occipital related abnormalities have been observed in a proportion of patients with schizophrenia,^40^ a subset of patients may display marked impairment on tasks relying on occipital cortex in addition to other regions that likely reflects a global deficit.

Patients in the deteriorated group displayed less marked deficits in executive function, memory, and language compared with the compromised group. However, they still presented with significant cognitive impairments relative to healthy controls, performing well below the normal range on tests of attention. Being a ‘hold’ measure in general, the WTAR is insensitive to factors that can produce cognitive decline, which is why it is used as a premorbid IQ estimate. Thus, it may be possible that some deteriorated patients did not really decline, but their performance-related deficits may have gone undetected given that the WTAR may be insensitive to them. However, early-performance-based deficits were unlikely to have gone undetected by the WTAR given that the deteriorated group displayed significantly more years of education, higher RBANS Attention scores, higher GAF scores, and a significantly lower percentage of unemployment at illness onset, a lower percentage having less than one friend and a significantly higher proportion who were employed in the past year than the compromised group. These significant differences between the deteriorated group and the compromised group would suggest that the deteriorated group did not simply have long-standing differential impairment in performance-related cognitive measures.

Patients in the preserved group performed in the normal range, with a small effect size compared with controls, in all cognitive domains. However, they did perform statistically significantly worse than healthy controls. Contrary to our hypotheses, the preserved group did not perform below the normal range on tests of executive function and attention. It is possible that the tests of executive function (i.e., COWAT and LNS) used in the current study may not be sensitive to the same aspects of prefrontal cortex function^41^ as the tests used previously^6^ although other studies have also not found evidence of executive function impairment,^8,20^ suggesting that the executive function deficit may not be universal in individuals with schizophrenia. Pantelis *et al*^42,43^ argue that earlier developing executive functions (such as attentional set-shifting) may be spared at illness onset, whereas more complex functions (such as spatial working memory) are susceptible to adolescent developmental disruptions and may be impaired at illness onset. They argue that early developing functions are susceptible to decline following onset and should be the target of early intervention.^44^ Overall, our results add to previous research suggesting there may be measureable cognitive decline in a substantial portion of patients.^2,6–8,19,20^

Patients in the preserved group displayed less functional impairment than those in the compromised group, consistent with previous research indicating that patients with intact cognitive functioning require less community support than other patients.^45^ Also consistent with other studies,^7,12^ patients in the preserved group displayed less-negative symptoms than patients in the compromised group. Intact executive function processes, the ability to plan and adapt, are necessary for functional independence and social integration.^41^ Considering that cognitive impairment and negative symptoms combine to contribute to functional outcome,^4^ interventions that can prevent decline of intact cognitive abilities may prevent deterioration in general functioning and should particularly focus on earliest stages of psychosis.^44^ The lack of significant differences between preserved and deteriorated groups in all but one of the negative symptoms is somewhat surprising given the observed connection between cognitive function and negative symptoms.^2,3^ Despite near intact cognitive function, a considerable proportion of patients in the preserved group reported social and occupational impairments. This suggests that factors other than those examined here, such as social cognition, impact on functional outcome as well,^46^ and these need to be considered when planning treatments to improve vocational outcomes.^47^

Limitations of our study include its cross-sectional nature. Although the WTAR is a validated measure of premorbid IQ,^48^ an actual premorbid IQ measure may more accurately classify patients, especially in cases of extreme scores.^49^ A validated current IQ measure would have aided comparison with previous studies. Exclusion of patients with IQs below 70 may have altered group proportions and means, not reflecting the number of patients with compromised intellectual function in the population. Level of education may have been a confounding factor. We did not control for education as it may mediate the relationship between premorbid and current IQ,^48^ and WTAR scores were significantly correlated with years of education in our sample, so removing the related variance from the analysis could have removed overlapping variance and obscured relevant relationships.^50^ The age and gender differences observed are a possible confound. However, the cognitive tests were adjusted for age and the only gender and group interaction was not among patient groups. Longitudinal assessment, or review of clinical notes may have enabled a more accurate assessment of negative symptoms than one-off scores on the SANS, which were generally low, indicating generally mild, or questionable negative symptoms in this cohort. In addition, positive symptom severity results were mixed, with reversed patterns of current versus lifetime results for delusions and hallucinations. These factors may be better addressed using a specific assay of positive symptoms.

In conclusion, these findings in a relatively large, community-based cohort confirm previous research demonstrating distinct neuropsychological profiles in schizophrenia and may provide further insight into the developmental processes involved. We also demonstrated the relevance of premorbid and current intellectual functioning to both negative symptoms and functional outcomes. The use of these profiles in future research may promote identification of the specific causes of deterioration in intellectual function by identifying other underlying and possibly biological factors which differentiate these patient groups.

## Figures and Tables

**Table 1:** Demographics characteristics of the samples of healthy adults and people with schizophrenia

	*Patients (*n*=534)*	*Control (*n*=635)*	*F/* χ^*2*^	P-*value*
Age (years)	39.2 (10.5)	41.9 (13.5)	14.7	**<0.001**
Education (years)	12.9 (2.9)	15.14 (3.1)	167.3	**<0.001**
Gender M/F ratio	66.5%/33.5%	44.7%/55.3%	55.4	**<0.001**

Means provided with s.d. in parentheses with the exception of gender for which percentages are provided. Bold font denotes large effect size, Cohen's d>0.80.

**Table 2:** Demographic characteristics of empirically clustered groups

	*CIQ (*n*=138)*	*DIQ (*n*=239)*	*PIQ (*n*=157)*	*HC (*n*=635)*	*F/χ*^*2*^	P	*CIQ<DIQ (p)*	*CIQ<PIQ (p)*	*DIQ<PIQ (p)*	*CIQ<HC (p)*	*DIQ<HC (p)*	*PIQ<HC (p)*
Age (years)	38.4 (9.7)	38.1 (10.1)	41.5 (11.4)	41.9 (13.5)	7.65	**<0.001**	0.808	**0.029**	**0.006**	**0.002**	**<0.001**	0.700
Education (years)	10.9 (2.4)	13.2 (2.4)	14.2 (2.8)	15.1 (3.1)	95.87	**<0.001**	**<0.001**	**<0.001**	**<0.001**	**<0.001**	**<0.001**	**<0.001**
Age of onset	23.2 (6.6)	22.7 (6.1)	24.4 (6.9)		3.44	**0.033**	0.395	0.128	**0.009**			
Illness duration	15.2 (9.5)	15.5 (9.5)	17.1 (10.7)		1.81	0.165	0.794	0.093	0.101			
Gender (M/F)	73.2%/26.8%	66.1%/33.9%	61.1%/38.9%	44.7%/55.3%	59.72	**<0.001**	0.094	**0.019**	0.184	**<0.001**	**<0.001**	**<0.001**

Abbreviations: CIQ, compromised group; DIQ, deteriorated group; F, female; HC, healthy control group; M, male; PIQ, preserved group.

Means provided with s.d. in parentheses with the exception of gender for which percentages are provided. Bold font denotes large effect size, Cohen's d>0.80.

**Table 3:** Comparison of performance on all cognitive tests on the basis of gender and the empirically derived cognitive subgroups of people with schizophrenia and healthy controls

*Cognitive Test*	*Gender*	*CIQ (*n*=138)*	*DIQ (*n*=239)*	*PIQ (*n*=157)*	*HC (*n*=635)*	*Group*		*Males*	*Females*	*Gender*		*Gender × Group*		*CIQ<DIQ*	*CIQ<PIQ*	*DIQ<PIQ*	*CIQ<HC*	*DIQ<HC*	*PIQ<HC*
						F	P			F	P	F	P	*d*	*d*	*d*	*d*	*d*	*d*
*Other cognitive tests*																			
Visuospatial construction	Male	−1.09 (1.0)	−0.58 (1.0)	−0.19 (1.0)	0.17 (1.0)	72.20	**<0.001**	−0.42 (1.1)	−0.72 (1.4)	16.17	**<0.001**	0.60	0.613	0.4	0.7	0.3	**1.0**	0.6	0.3^a^
	Female	−1.56 (0.8)	−0.87 (1.0)	−0.32 (1.1)	−0.14 (1.0)														
Language	Male	−1.51 (0.9)	−1.19 (0.8)	−0.88 (0.8)	−0.28 (1.0)	111.04	**<0.001**	−0.96 (1.0)	−0.53 (1.3)	40.45	<0.001	0.69	0.557	0.3	0.6	0.3	**1.1**	**0.9**	0.6
	Female	−1.25 (0.9)	−0.74 (0.9)	−0.40 (0.9)	0.26 (0.9)														
Delayed verbal memory	Male	−2.65 (1.5)	−2.00 (1.5)	−0.63 (1.1)	−0.01 (1.0)	219.73	**<0.001**	−1.32 (1.2)	−0.88 (1.6)	28.19	<0.001	4.18	0.006	0.4	**1.2**	**0.8**	**1.6**	**1.2**	0.5
	Female	−2.06 (1.3)	−1.35 (1.0)	−0.19 (1.0)	0.08 (0.9)														
Verbal fluency	Male	−1.25 (0.8)	−0.84 (0.8)	−0.32 (0.9)	−0.07 (0.9)	90.62	**<0.001**	−0.62 (0.9)	−0.52 (1.1)	2.40	0.122	0.13	0.941	0.4	**0.9**	0.5	**1.2**	**0.8**	0.2
	Female	−1.17 (0.8)	−0.69 (0.9)	−0.28 (0.8)	0.04 (0.8)														
																			
*Clustering Variables*																			
WTAR	Male	−2.66 (1.0)	−0.21 (0.7)	0.00 (0.9)	−0.02 (1.1)	255.91	**<0.001**	−0.72 (1.0)	−0.71 (1.3)	0.04	0.834	0.87	0.454	**2.2**	**2.2**	0.2	**2.1**	0.2	<0.1^a^
	Female	−2.63 (1.0)	−0.36 (0.8)	0.14 (0.8)	0.02 (0.9)														
Immediate memory	Male	−2.55 (0.9)	−1.92 (0.9)	−0.53 (0.8)	−0.13 (1.0)	331.68	**<0.001**	−1.28 (0.1)	−0.96 (0.1)	22.63	**<0.001**	0.69	0.560	0.6	**1.9**	**1.3**	**2.1**	**1.6**	0.4
	Female	−2.26 (0.8)	−1.53 (0.9)	−0.12 (0.8)	0.09 (1.0)														
Attention	Male	−2.42 (0.8)	−1.68 (0.8)	−0.47 (0.7)	−0.06 (1.0)	314.83	**<0.001**	−1.16 (1.0)	−1.01 (1.3)	4.73	**0.030**	0.19	0.904	**0.8**	**2.1**	**1.3**	**2.2**	**1.5**	0.4
	Female	−2.25 (0.8)	−1.58 (0.7)	−0.25 (0.8)	0.05 (1.0)														
Letter number sequencing	Male	−1.77 (0.8)	−1.28 (0.7)	−0.04 (0.8)	0.04 (1.0)	206.89	**<0.001**	−0.77 (1.0)	−0.83 (1.3)	0.88	0.349	0.13	0.940	0.5	**1.8**	**1.4**	**1.7**	**1.3**	0.1^a^
	Female	−1.87 (0.8)	−1.28 (0.6)	−0.12 (0.8)	−0.04 (1.0)														

Abbreviations: CIQ, compromised group; DIQ, deteriorated group; PIQ, preserved group; HC, healthy control group; RBANS, The Repeatable Battery for the Assessment of Neuropsychological Status.

RBANS index and other cognitive scores were converted to *z*-scores (relative to healthy control means and s.d.). Means with s.d. in parentheses provided. All tests significant after Bonferroni correction *P*<0.002 to *P*=0.05 unless indicated.

Visuospatial Constructional=RBANS visuospatial/constructional Index; Language=RBANS language index; Delayed verbal memory=RBANS delayed memory Index. Verbal Fluency=Controlled Oral Word Association Test

a Indicates pairwise comparison was not significant. *d* indicates Cohen's *d* effect size (large effects >0.8 in bold).

**Table 4:** Comparison of symptom severity and general functioning on the basis of empirically derived cognitive subgroups of people with schizophrenia

	*CIQ (*n*=131)*	*DIQ (*n*=228)*	*PIQ (*n*=153)*	F	P	*CIQ versus DIQ (d)*	*CIQ versus PIQ (d)*	*DIQ versus PIQ (d)*
*Negative symptoms*	*Median* (s.d.)	*Median* (s.d.)	*Median* (s.d.)					
*Avolition–apathy*								
Global avolition–apathy	2.3 (1.4)	1.9 (1.4)	1.7 (1.4)	7.07	**0.001**	0.2	0.4^a^	0.2
Impersistence at work/school	2.8 (1.6)	2.4 (1.6)	2.1 (1.6)	7.66	**0.001**	0.2	0.4^a^	0.2
Physical anergia	1.6 (1.4)	1.3 (1.4)	0.8 (1.4)	11.20	**<0.001**	0.2	0.5^a^	0.3^a^
Grooming	1.2 (1.3)	0.8 (1.3)	0.9 (1.3)	3.27	0.039			
								
*Affective flattening*								
Global affective flattening	1.9 (1.4)	1.7 (1.4)	1.4 (1.4)	4.41	**0.009**	0.1	0.3^a^	0.2
Facial expression	1.9 (1.5)	1.9 (1.5)	1.6 (1.5)	2.1	0.123			
Spontaneous movement	0.8 (1.1)	0.7 (1.1)	0.6 (1.1)	1.58	0.206			
Gestures	1.4 (1.3)	1.2 (1.3)	0.9 (1.3)	4.57	0.011			
Eye contact	1.1 (1.4)	1.1 (1.4)	1.0 (1.4)	0.55	0.574			
Non-responsivity	0.9 (1.2)	0.8 (1.2)	0.5 (1.2)	2.96	0.053			
Inappropriate	0.5 (0.9)	0.3 (0.9)	0.3 (0.9)	2.39	0.093			
Vocal inflections	1.6 (1.4)	1.3 (1.4)	1.1 (1.4)	4.73	**0.009**	0.2	0.2^a^	0.2
								
*Anhedonia–asociality*								
Global asociality	2.5 (1.4)	2.1 (1.4)	1.9 (1.4)	6.11	**0.002**	0.2	0.3^a^	0.1
Recreational	2.1 (1.5)	1.8 (1.5)	1.5 (1.5)	5.84	**0.003**	0.1	0.3^a^	0.2
Sexual activity	1.6 (1.6)	1.3 (1.6)	1.0 (1.6)	4.89	**0.008**	0.2	0.3^a^	0.1
Intimacy	2.0 (1.6)	1.7 (1.6)	1.4 (1.6)	5.15	**0.006**	0.2	0.3^a^	0.1
Relationships	2.4 (1.6)	2.0 (1.6)	1.9 (1.6)	4.85	**0.008**	0.2	0.3^a^	0.1
								
*Alogia*								
Global alogia	1.3 (1.3)	1.3 (1.3)	1.1 (1.3)	1.25	0.289			
Poverty of speech	1.1 (1.3)	0.7 (1.3)	0.7 (1.3)	4.39	0.013			
Speech content	0.8 (1.3)	0.9 (1.3)	0.8 (1.3)	0.48	0.618			
Blocking	0.4 (0.8)	0.3 (0.8)	0.2 (0.8)	1.01	0.365			
Response latency	0.6 (1.1)	0.5 (1.1)	0.5 (1.1)	0.67	0.511			
								
*Positive symptoms*	*Median (s.d.)*	*Median (s.d.)*	*Median (s.d.)*	*χ^2^*	P	P	P	P
*Hallucinations*								
Lifetime	2.9 (2.1)	3.0 (2.3)	3.0 (2.5)	0.02	0.990			
Past week	0.9 (1.8)	0.6 (1.8)	0.4 (1.3)	12.11	**0.002**	0.197	**0.002**	0.125
								
*Delusions*								
Lifetime	4.3 (2.7)	5.1 (2.4)	5.4 (2.7)	8.86	**0.012**	0.078	**0.011**	0.991
Past week	0.9 (2.3)	0.7 (1.8)	0.5 (2.4)	5.106	0.078			

*General functioning*	*%*	*%*	*%*	χ*^2^*	P	P	P	P
% Never married	44%	55%	49%	4.61	0.100			
% Brain disorder prior to onset	7%	2%	5%	6.24	0.044			
% Unemployed at onset	36%	20%	19%	15.80	**<0.001**	**<0.001**	**0.001**	0.915
% Poor premorbid work adjustment	31%	30%	22%	4.36	0.113			
% Poor premorbid social adjustment	39%	33%	32%	1.81	0.404			
								
*Course of Illness*								
% Acute onset	32%	25%	34%	3.97	0.137			
% Chronic course	51%	37%	30%	14.74	**0.001**	0.007	**<0.001**	0.134
*Current Functioning*								
% ⩽1 Friend	41%	24%	20%	18.13	**<0.001**	**0.001**	**<0.001**	0.353
% Employed in last year	19%	41%	41%	20.85	**<0.001**	**<0.001**	**<0.001**	0.978
% Obvious dysfunction in self care	28%	24%	20%	6.80	0.558			
								
*Global Functioning*	*Mean (s.d.)*	*Mean (s.d.)*	*Mean (s.d.)*	F	P	P	P	P
GAF^a^	44.7 (11.4)	52.7 (11.5)	55.6 (12.6)	32.44	**<0.001**	**<0.001**	**<0.001**	0.015

Abbreviations: CIQ, Compromised group; DIP, diagnostic interview for psychosis; DIQ, Deteriorated group; GAF, Global Assessment of Functioning; PIQ, preserved group; SANS, Scale for Assessment of Negative Symptoms.

Means with standard deviation in parentheses provided for negative symptoms. Medians and s.d. are presented for positive symptoms. Negative symptoms from SANS. Cohen’s *d* effect sizes are shown for negative symptoms pairwise comparisons. For SANS results.

a Indicates significant result after Bonferroni correction. For general function variables (consisting of demographic factors and items 9, 13–15, and 17 from the DIP, the percentage of participants in each group endorsing each item are presented, except for GAF where means with s.d. in parentheses are presented. Bold font denotes large effect size, Choen's d>0.80.
