# Supplementary material for: The impact of premorbid and current intellect in schizophrenia: cognitive, symptom, and functional outcomes
Source: NPJ Schizophr. 2015 Nov 4;1:15043–. doi: 10.1038/npjschz.2015.43 (PMC4849463; doi:10.1038/npjschz.2015.43)
Supplement: Supplementary Information [file npjschz201543-s1.pdf]

Supplemental Material

The Relationship between Pre-morbid and Current Intellect Estimates in Schizophrenia: Cognitive, Symptom and Functional Status

Running Title: Pre-morbid and Current Intellect in Schizophrenia

Ruth Wells [a,b], Vaidy Swaminathan [c, d, e, f], Suresh Sundram [c, d, f, g], Danielle Weinberg [a,b], Jason Bruggemann [a, b], Isabella Jacomb [b], Vanessa Cropley [c], Rhoshel Lenroot [a, b, e], Avril M. Pereira [c, f], Andrew Zalesky [c], Chad Bousman [c], Christos Pantelis [c, e], Cynthia Shannon Weickert [a, b, e], Thomas W. Weickert [a, b, d, *]

[a] School of Psychiatry, University of New South Wales, Sydney, NSW, Australia
[b] Neuroscience Research Australia, Randwick, Sydney, NSW, Australia
[c] Department of Psychiatry, University of Melbourne, Parkville, Victoria, Australia
[d] Northern Psychiatry Research Centre, North Western Mental Health, Melbourne Health, Victoria, Australia
[e] Schizophrenia Research Institute, Sydney, NSW, Australia
[f] Molecular Psychopharmacology Laboratory, The Florey Institute of Neuroscience and Mental Health, Parkville, Victoria, Australia

[g] Current address: Department of Psychiatry, School of Clinical Sciences, Monash University, Clayton, Victoria, Australia

* Corresponding Author

Corresponding Author Address:
Thomas Weickert, PhD
Neuroscience Research Australia
Barker Street
Randwick, NSW 2031
Office: +61 02 9399 1730
Fax: +61 02 9399 1034
email: t.weickert@unsw.edu.au

Supplemental Material

Methods

Clinically Derived Groups Method

To determine whether the resulting empirical groups were in agreement with clinically meaningful criteria, patients were also categorized into three groups based on their decline from pre-morbid IQ estimate (WTAR). A composite current global cognitive ability estimate was created by averaging the WAIS-III LNS and RBANS Attention and Immediate Memory z-scores, with z-scores below negative 1 indicating performance more than one standard deviation below control means, and therefore below the normal range. As per Weickert and colleagues[6] patients were allocated to: (1) the *preserved group*, WTAR scores ≥ 90, and current global cognitive ability z-score > – 1 ; (2) the *deteriorated group,* WTAR scores ≥ 90 and current global cognitive ability z-score < – 1 ; or (3) the *compromised group,* WTAR score < 90 and current global cognitive ability z-score < – 1.

Supplemental Table S1. Frequency of antipsychotics prescribed in the previous month to patients with schizophrenia in each of the empirically derived subgroups.

Medication	CIQ	DIQ	PIQ	Total
No antipsychotics	8	28	28	64
amisulpride	7	10	14	31
amisulpride & aripiprazole	2	1	2	5
amisulpride & aripiprazole & olanzapine & pericyazine & quetiapine fumerate & risperidone	0	1	0	1
amisulpride & chlorpromazine hydrochloride	0	1	0	1
amisulpride & chlorpromazine hydrochloride & clozapine	0	1	0	1
amisulpride & clozapine	3	7	3	13
amisulpride & clozapine & olanzapine	0	0	1	1
amisulpride & clozapine & quetiapine fumerate	0	1	0	1
amisulpride & flupenthixol decanoate	0	1	1	2
amisulpride & olanzapine	2	1	0	3
amisulpride & quetiapine fumerate	1	3	0	4
amisulpride & quetiapine fumerate & trifluoperazine hydrochloride	0	0	1	1
amisulpride & risperidone	1	0	0	1
amisulpride & risperidone consta	0	2	0	2
amisulpride & trifluoperazine hydrochloride	1	0	0	1
amisulpride & ziprasidone hydrochloride	0	1	1	2
aripiprazole	6	15	9	30
aripiprazole & clozapine	2	1	1	4
aripiprazole & olanzapine	1	3	1	5
aripiprazole & quetiapine fumerate	1	2	2	5
aripiprazole & risperidone consta	1	0	0	1
chlorpromazine hydrochloride	1	0	0	1
chlorpromazine hydrochloride & flupenthixol decanoate	1	0	0	1
chlorpromazine hydrochloride & olanzapine	0	0	1	1
chlorpromazine hydrochloride & risperidone & risperidone consta	1	0	0	1
chlorpromazine hydrochloride & thiothixene & olanzapine	0	0	1	1

clozapine	35	31	8	74
clozapine & amisulpride	0	1	0	1
clozapine & flupenthixol decanoate	0	0	1	1
clozapine & olanzapine	1	0	0	1
clozapine & quetiapine fumerate	0	2	0	2
clozapine & risperidone	1	2	0	3
clozapine & risperidone consta	0	2	1	3
clozapine & ziprasidone hydrochloride	1	1	0	2
clozapine & zuclopenthixol decanoate	1	1	0	2
flupenthixol decanoate	4	2	4	10
flupenthixol decanoate & olanzapine	2	1	0	3
flupenthixol decanoate & quetiapine fumerate	1	0	0	1
fluphenazine decanoate	1	1	0	2
fluphenazine decanoate & olanzapine	0	1	1	2
fluphenazine decanoate & quetiapine fumerate	0	1	0	1
fluphenazine decanoate & quetiapine fumerate & trifluoperazine hydrochloride	0	1	0	1
fluphenazine decanoate & thioridazine hydrochloride & olanzapine	0	0	1	1
haloperidol	0	3	2	5
haloperidol & olanzapine & ziprasidone hydrochloride	1	0	0	1
haloperidol & quetiapine fumerate	0	1	0	1
haloperidol decanoate	0	2	0	2
olanzapine	15	36	23	74
olanzapine & flupenthixol decanoate	1	0	0	1
olanzapine & quetiapine fumerate	2	3	0	5
olanzapine & quetiapine fumerate & risperidone	0	1	0	1
olanzapine & quetiapine fumerate & risperidone & thioridazine hydrochloride	0	1	0	1
olanzapine & risperidone	1	0	3	4
olanzapine & risperidone consta	1	3	0	4
olanzapine & ziprasidone hydrochloride	1	0	0	1
olanzapine & zuclopenthixol decanoate	2	1	1	4
pericyazine	0	2	0	2
quetiapine fumerate	7	16	14	37

quetiapine fumerate & risperidone	2	1	1	4
quetiapine fumerate & risperidone consta	1	0	1	2
quetiapine fumerate & trifluoperazine hydrochloride	0	1	2	3
quetiapine fumerate & ziprasidone hydrochloride	0	1	0	1
quetiapine fumerate & zuclopenthixol acetate	0	1	0	1
quetiapine fumerate & zuclopenthixol decanoate	1	0	0	1
risperidone	8	20	13	41
risperidone & risperidone consta	0	2	1	3
risperidone & trifluoperazine hydrochloride & ziprasidone hydrochloride	0	0	1	1
risperidone consta	5	5	7	17
trifluoperazine hydrochloride	0	3	3	6
ziprasidone hydrochloride	1	6	1	8
ziprasidone hydrochloride & zuclopenthixol decanoate	0	1	0	1
zuclopenthixol decanoate	3	3	1	7
zuclopenthixol dihydrochloride	0	0	1	1
**Total**	**138**	**239**	**157**	**534**

Number of patients in each group on each antipsychotic medication, or combination of antipsychotic medications.

Supplemental Table S2. Demographic Characteristics of Clinically Derived Groups

	**CIQ (n=147)**	**DIQ (n=227)**	**PIQ (n=160)**	**HC (n=635)**	F/χ^2	***p***	**CIQ < DIQ (*p*)**	**CIQ < PIQ (p)**	**DIQ < PIQ *(p)***	**CIQ < HC (*p*)**	**DIQ < HC *(p)***	**PIQ < HC *(p)***
Age (years)	39.6 (10.0)	37.8 (10.0)	40.8 (11.3)	41.9 (13.5)	6.91	**<0.001**	<0.163	0.379	**0.016**	**0.036**	**<0.001**	0.298
Education (years)	11.0 (2.5)	13.1 (2.5)	14.3 (2.7)	15.1 (3.1)	97.67	**<0.001**	**<0.001**	**<0.001**	**<0.001**	**<0.001**	**<0.001**	**0.001**
Age of Onset	23.8 (7.0)	22.4 (6.0)	24.1 (6.7)		3.71	**0.025**	**0.047**	0.676	**0.013**			
Illness Duration	15.8 (9.6)	15.4 (9.6)	16.7 (10.5)		0.88	0.416	0.680	0.419	0.188			
Gender (M/F)	70.7%/29.3%	67.0%/33.0%	61.9%/38.1%	44.7%/55.3%	57.87	**<0.001**	0.411	0.101	0.302	**<0.001**	**<0.001**	**<0.001**

Notes: Means provided with standard deviation in parentheses with the exception of gender where percentages are provided. CIQ: Compromised group; DIQ: Deteriorated group; PIQ: Preserved group; HC: Healthy control group.

Supplemental Table S3. Clinically Derived Group Performance on Cognitive Tests

Cognitive Test	CIQ (n=147)	DIQ (n=227)	PIQ (n=160)	HC (n=635)	Group *F/χ2*	*p*	CIQ < DIQ *d*	CIQ < PIQ *d*	DIQ < PIQ *d*	CIQ < HC *d*	DIQ < HC *d*	PIQ < HC *d*
**Visuospatial Constructional**	-1.2 (0.9)	-0.7 (1.0)	-0.2 (1.0)	0.0 (1.0)	252.35	**<0.001**	0.4	**0.8**	0.4	**1**	0.6	0.2[a]
**Language** [b]	-1.4 (0.9)	-1.2 (0.9)	-0.6 (0.9)	-.15 (1.0)	300.64	**<0.001**	0.2[a]	0.6	0.4	**1.3**	**1.1**	**0.8**[a]
**Delayed Verbal Memory** [b]	-1.8 (1.5)	-1.5 (1.4)	-0.4 (1.1)	.02 (1.0)	474.05	**<0.001**	0.2	**1.1**	**0.8**	**1.6**	**1.4**	0.7
**Verbal Fluency**	-1.2 (0.8)	-0.8 (0.8)	-0.3 (0.8)	0 (0.86)	108.63	**<0.001**	0.3	**0.9**	0.5	**1.2**	**0.8**	0.3

Notes: Means with standard deviation in parentheses provided. All tests significant after Bonferroni correction $p < 0.002$ to p = .05 unless indicated. [a] Indicates pairwise comparison is not significant. *d* indicates Cohen's d effect size (large effects > 0.8 in bold. CIQ = Compromised group; DIQ = Deteriorated group; PIQ = Preserved group; HC = Healthy control group. Visuospatial / Constructional = The Repeatable Battery for the Assessment of Neuropsychological Status (RBANS) visuospatial/ constructional Index; Language = RBANS language index; Delayed verbal memory = RBANS delayed memory subtest. Verbal Fluency = Controlled Oral Word Association Test. [b] Indicates medians and Kruskal-Wallis Test performed due to non-normal distributions.

Supplemental Figure S1. Scatter Plots of Current Cognitive Function Variables and WTAR displaying Individuals in each Group

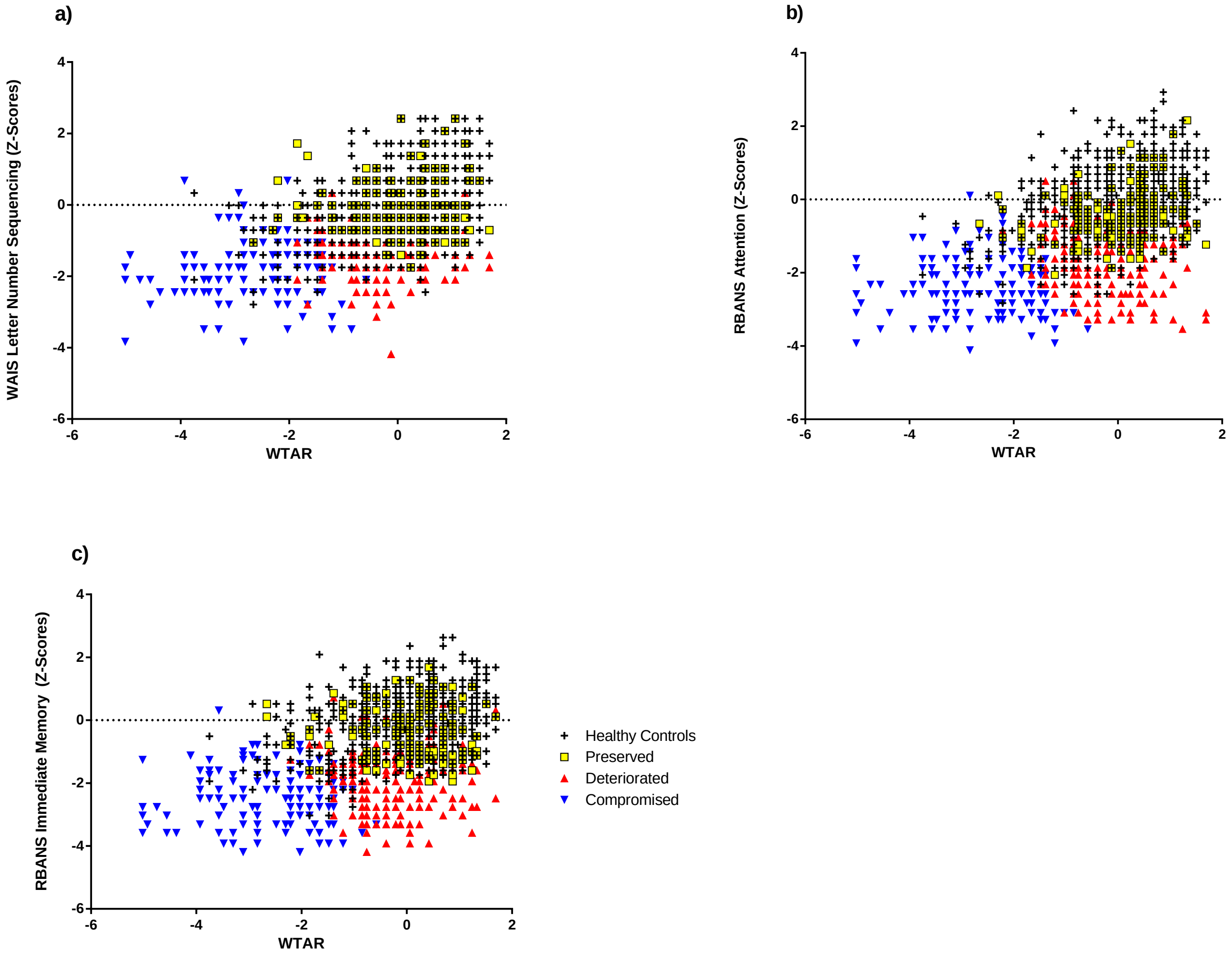

Scatter plots display data points for each individual comparing current cognitive function variables to an estimate of pre-morbid IQ (WTAR). Individuals from each group of the empirical clustering procedure can be observed to cluster together. The compromised group showing low current and low pre-morbid scores; the deteriorated group showing low current and average-high pre-morbid scores; the preserved group showing average-high scores on both current and pre-morbid measures. WTAR, Wechsler Test of Adult Reading**.** a) Letter Number Sequencing subtest (WAIS-III) and WTAR. b) Attention: Attention subtest of RBANS and WTAR. c) Immediate Memory: Immediate memory subtest of RBANS and WTAR.
